# Incidence and Clinical Risk Factors of Post-Operative Complications following Primary Total Hip Arthroplasty: A 10-Year Population-Based Cohort Study

**DOI:** 10.3390/jcm13010160

**Published:** 2023-12-27

**Authors:** Yen-Sheng Lin, Joshua J. DeClercq, Gregory D. Ayers, Ruby J. Gilmor, Garen Collett, Nitin B. Jain

**Affiliations:** 1 Department of Orthopaedic Surgery, University of Texas Southwestern, Dallas, TX 75390, USA; yen-sheng.lin@utsouthwestern.edu (Y.-S.L.); garen.collett@utsouthwestern.edu (G.C.); 2 Department of Physical Medicine and Rehabilitation, University of Texas Southwestern, Dallas, TX 75390, USA; 3 Department of Physical Medicine and Rehabilitation, Vanderbilt University Medical Center, Nashville, TN 37212, USAdan.ayers@vumc.org (G.D.A.); 4 Sinai Hospital of Baltimore, Baltimore, MD 21215, USA; 5 Department of Population & Data Sciences, University of Texas Southwestern, Dallas, TX 75390, USA

**Keywords:** total hip arthroplasty, complications, state inpatient database

## Abstract

**Background:** Total hip arthroplasty (THA) has become a growing treatment procedure for debilitating hip pathologies. Patients experienced post-operative complications and revision surgeries according to large THA registries. To fully understand the short-term and long-term post-operative outcomes following THA, the purpose of this study is to examine the incidence of post-operative complications following primary THA and to examine how this trend has changed over 10 years within community hospitals in the US using large databases. **Methods:** This study queried the State Inpatient Database (SID) for primary THA between 2006 and 2015. Individual patients were followed forward in time until the first instance of a post-operative complication. The multivariable logistic regression analyses were computed to examine which post-operative complications were independent predictors of pre-operative comorbidities. **Results:** Median age of patients was 67 years, and 56% of patients were female. Females with avascular necrosis (AVN) as an indication for THA had a 27% higher risk of complication. Females with osteoarthritis (OA) as an indication for THA had a 6% higher risk of complication. Post-operative complications occurred with higher frequencies in the first two months of THA and the highest risks of THA complications within the first 6 months. **Conclusion:** The most common indication is OA in elders with primary THA. Females and those of black ethnicity showed the greatest risks of THA complications. Data from our large study can be used to understand post-operative complications and readmissions after THA. Our study also provides data on risk factors associated with these complications.

## 1. Introduction

The hip is the third most common site of osteoarthritis (OA) in the United States (US) [1]. The medical cost of OA has been estimated to account for 1–2.5% of the gross domestic product in various high-income countries [2]. Total hip arthroplasty (THA) is the definitive procedure for primary hip OA and has become a more popular treatment option for debilitating hip pathologies over the years. More than 500,000 primary THAs were performed in 2020, and it is projected that the annual hospital cost of THA will be USD 753.4 million by 2030 [3,4]. In the US, the numbers are steadily growing due to population aging, as well as the increasing prevalence of obesity and a high body mass index [1,5,6]. Advancements have been made in the implant material, design of surgical techniques, and perioperative management in recent decades [7]. Although THA is a highly successful procedure with a low complication rate, it is also one of the most common reasons for revision and subsequent re-revision surgeries according to large THA registries [8]. Prior studies have reported complication rates in patients after THA [5,9]. However, these studies were limited to information from a single hospital or geographical location and did not consider comorbidities that affected post-operative outcomes. The continued examination of post-operative complications is warranted, particularly in those who have the highest risks of post-operative complications to guide personalized and targeted perioperative cares. The purpose of this study is to examine the incidence of post-operative complications following primary THA and to examine how this trend has changed over 10 years within community hospitals in the US using large databases. The secondary objective is to determine the predictive factors for post-operative complications after primary THA. We hypothesized differential hazards according to the indication for surgery based on age, gender, and race.

## 2. Methods

### 2.1. Data Source

This study queried the State Inpatient Database (SID) for all patients that underwent primary THA. Comorbidities, post-operative complications, and post-operative comorbidities were determined based on prespecified diagnosis codes after the index THA using International Classification of Disease, Ninth Revision (ICD-9) codes between 2006 and 2015 (See Appendix A for procedure code 81.51 used to identify patients who underwent THA). This SID database contains 100% of inpatient procedures of individual states and has been increasingly used in orthopedic research in recent decades [10]. The SID comprises a set of longitudinal state-specific databases, consisting of over 2.1 million observations of patients within the same state–year combinations that contain information on all inpatient stays in a given state and year. Data from a total of 10 states (Arizona, California, Florida, Iowa, Maryland, North Carolina, Nebraska, New Mexico, New York, and Utah) were available for analysis from 2006 to 2015 [11]. The SID were previously used to examine treatment outcomes, costs, and trends in hip, knee, and shoulder arthroplasty [12,13,14]. Only complications requiring in-patient admissions were included in our study, since this study was based on in-patient databases. Codes for complications were further grouped into the following categories: mechanical complications, infection due to prosthetic devices, and thromboembolism or other complications. Methods and approaches to achieve the de-identification of patient information were in accordance and compliance with the Health Insurance Portability and Accountability Act [15].

### 2.2. Study Population and Patient Sample

Patients that underwent primary THA during the period specified above, who also maintained at least one year of post-operative follow-up in the SID database, were included. Patients who did not have medical records at least one year prior to and one year after primary THA were excluded from this study. Post-operative complications were modeled as survival data, in which the first THA that appeared on a patient’s record was considered as the index procedure. Individual patients were followed forward in time until the first instance of a complication (for each category, as well as for any complication) or until contiguous state-year data were no longer available for the state in which patients had their index procedure.

### 2.3. Demographics, Clinical, and Outcome Measures

Patient demographics included the age upon admission for the initial THA procedure, gender, race, a three-level urban-rural designation for the patient’s county of residence, hospital length of stay (LOS), a ZIP-code-based quartile classification for the estimated household median income of residents, and the Elixhauser comorbidity readmission score. Race was categorized as white, black, Hispanic, and other. The Elixhauser Comorbidity readmission score was derived from the HCUP Elixhauser Comorbidity v2023.1 software [16]. Even though the score was designed to predict 30-day readmissions, it was used here as a surrogate variable for a predictor of any complications. Indications for THA surgery were based on the primary diagnosis code associated with the THA procedure, including OA, avascular necrosis (AVN), trauma, and others. Patients were followed longitudinally through a unique visit-linking identifier variable, which was provided along with the time between inpatient visits. Patients were followed forward in time until data were no longer available for that state. For patients presenting more than one THA procedures, the initial incidence was used as the index procedure.

### 2.4. Statistical Analyses

Descriptive statistics were used to summarize the population. Means and standard deviations (SD), or medians and interquartile ranges (IQRs), were used for continuous variables. Frequencies and proportions were used for categorical variables. The probability of post-operative complications was estimated by taking the complement of the Kaplan–Meier survival curve to estimate the probability of hip complications. Data imputation was performed via the predictive mean matching method, which used the impute function in the RMS R-package [17]. The primary outcome of time to the post-operative complication and each subgroup analysis were modeled using the Cox proportional hazards (PH) model [18]. The admission date was provided in either months or quarters depending on the state and year. Admission dates were placed at the midpoint for the particular time period. To examine the post-operative complications that were independent predictors of pre-operative comorbidities, multivariable logistic regression analyses were computed. Continuous predictors of a complication (LOS, age, and readmission score) were modeled using restricted cubic splines with five knots to allow for nonlinear associations with the outcome [19]. Other predictors in the models included gender, race, urban–rural designation, ZIP-code-based income quartile, and indication for surgery. Only post-operative complications that were deemed statistically significant based on test results were included in the regression model. Alpha level < 0.05 was considered as statistically significant for all tests.

## 3. Results

Among the 540,097 patients who underwent THA, 35,005 had an in-patient admission due to complications. The median (IQR) age of patients was 67 years, and 56% of patients were female. The indications for THA included OA (85.6% of patients) and trauma (6.2% of patients). Among the 35,005 patients reporting complications, 18,506 (52.9%) had a mechanical complication, 10,881 (31.1%) had a thromboembolism, 5493 (15.7%) had an infection due to the prosthetic device, and 3613 (10.3%) had other complications. Table 1 illustrates the number of patients experiencing any post-operative complications that required surgery, and Table 2 shows the patients’ demographic information and indications for primary THA.

**Figure 1:**
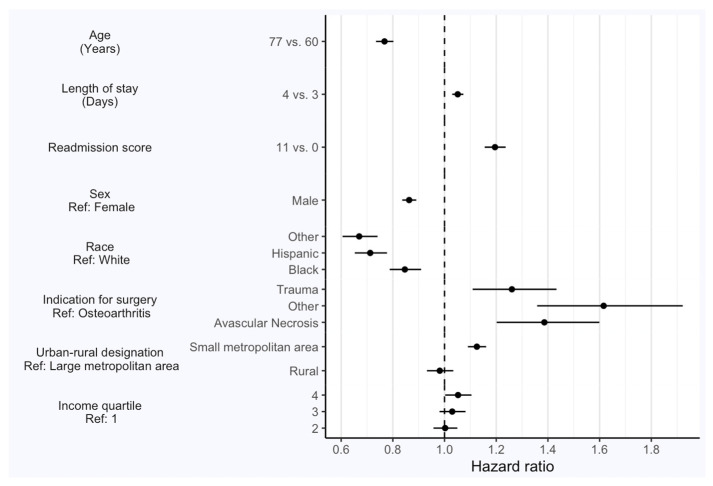
Univariate predictors of hazard ratios (HRs) for complications after total hip arthroplasty (THA) including age, length of stay, readmission score, sex, race, indication for surgery, urban–rural designation, and income quartile.

In univariate analysis, the age, length of stay, readmission score, gender, race, indication for surgery, as well as urban–rural designation were all significantly associated with complications after THA (Figure 1). Male patients were less likely to have THA complications than female patients. The Elixhauser 30-day readmission score was also a very strong nonlinear predictor of any complications (HR 1.20, 95% CI 1.16–1.24). Patients residing in small metropolitan areas were more likely to have complications than those in large metro areas (HR 1.13, 95% CI 1.09–1.16). Meanwhile, patients in rural areas were less likely to experience complications than those in large metro areas (HR 0.98, 95% CI 0.93–1.03).

**Figure 2:**
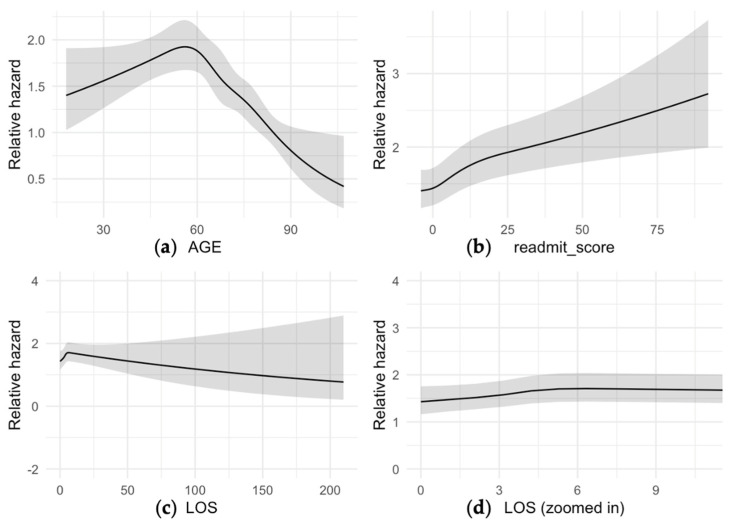
Relative hazard curves of 20,611 patients and the relationship between continuous variables including age (**a**), Elixhauser 30-day readmission score (readmit_score) (**b**), and length of stays (LOS; inpatient days) (**c**,**d**) as predictors of post-operative complications within the first 12 months after THA. Each line and shadow represented the mean and standard deviation.

**Figure 3:**
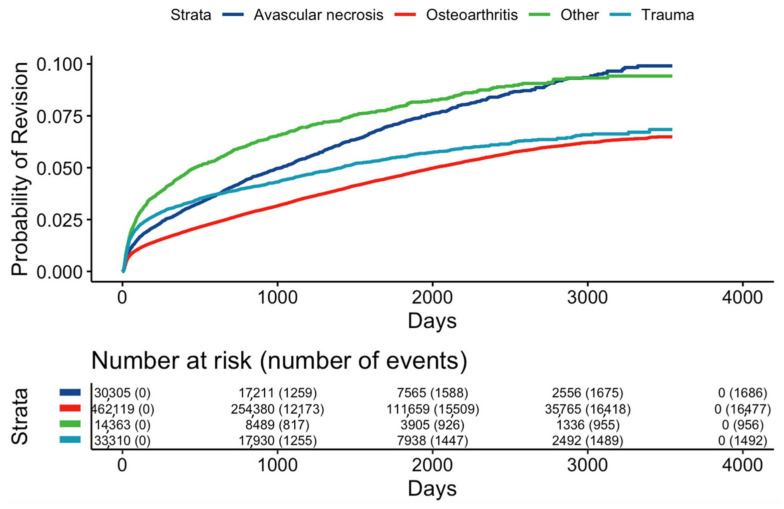
Revision survival curves of indications following total hip arthroplasty.

Figure 2 represents the relative hazard of post-operative complications within three months after primary THA. The relative risk was the highest with 1.8 at the age of 55 (Figure 2a). The higher Elixhauser readmission scores were strong predictors of post-operative complications (Figure 2b). The relative risk also increased in the first four days (Figure 2c) but declined one week after primary THA (Figure 2d). We found evidence of a nonlinear association between the hospital length of stay and the outcome (*p* < 0.001), with an increasing hazard from 0 to 3 days, followed by a near level-out of the hazard curve. Indications for the 10-year probability of post-operative complications were non-traumatic AVN, OA, and trauma (Figure 3).

Indications during readmissions were shown by the highest success rates and lowest risks for OA within the first three years, followed by trauma and AVN. The vascular causes represented the third most frequent indication for surgery within the first year of THA. However, the readmission rate of non-traumatic AVN suppressed and continued to outweigh readmissions due to trauma after two years. Within the first three months post-THA, the hazard of having a post-operative complication was the highest. Subsequently, the hazard for complications remained relatively constant over time. The one-year, five-year, and ten-year survival rates of THA were 0.969 (95% confidence interval (CI) 0.969–0.970), 0.917 (95% CI 0.916–0.918), and 0.875 (95% CI 0.873–0.877), respectively. In the Cox PH model for any complications, we found strong evidence of a nonlinear association between age and the outcome (*p* < 0.001). This relationship also had an interaction with the indication for surgery (*p* < 0.001).

**Figure 4:**
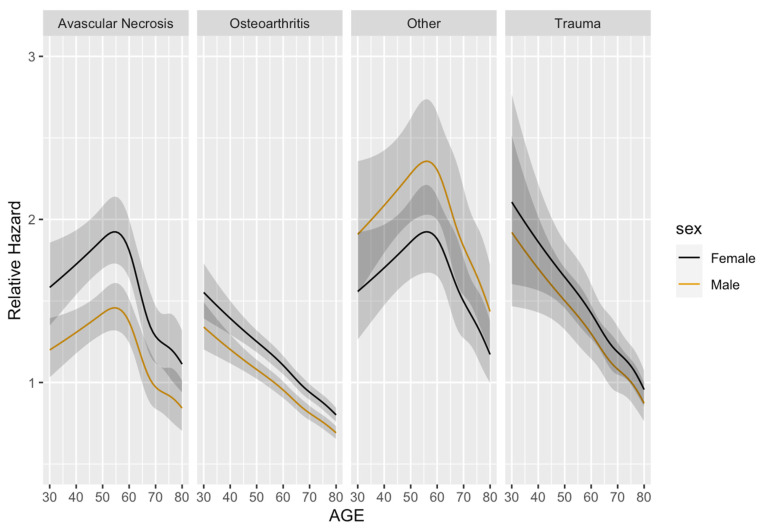
Relative hazards of post-operative complications from avascular necrosis, osteoarthritis, trauma, and others, as well as the interaction with age, from the age of 30–80 between 2006 and 2015.

**Figure 5:**
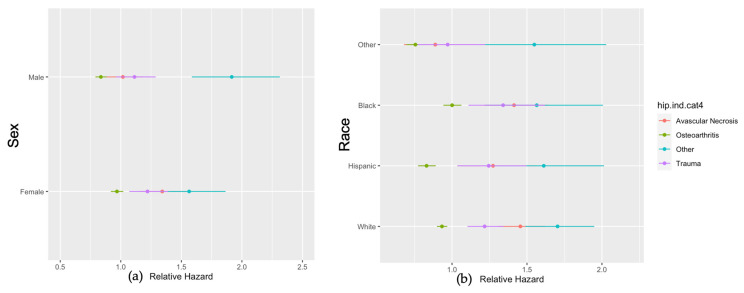
Relative hazards of THA complications from avascular necrosis, osteoarthritis, trauma, and others, as well as the interaction with gender (**a**) and race (**b**) between 2006 and 2015.

**Figure 6:**
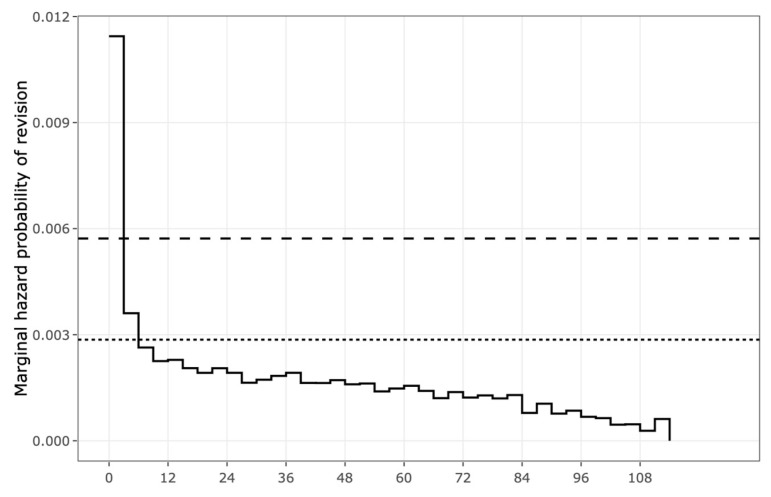
Crude hazard rates of probability for post-operative revision over 10 years, presenting the highest risks of post-operative revision within the first 6 months. Dash and dot lines represented the probability of revision dropped 50% from 3 to 6 months.

**Figure 7:**
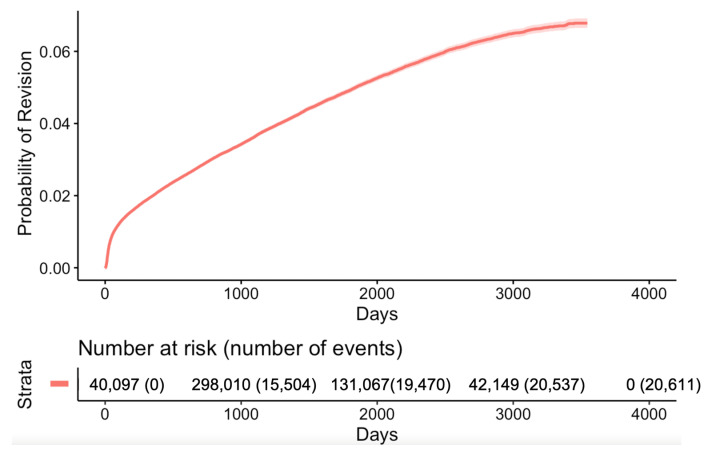
Kaplan–Meier survival curve for all hip complications between 2006 and 2015.

Figure 4 shows the difference in the relative hazards of post-operative complications across the range of patient ages and gender between 2006 and 2015, stratified by the indication for surgery without any additional factors included. The relative hazard peak of AVN was at the age of 55, showing the highest complication rate. However, there were visible peaks in OA and trauma at the age of 30. The peaks of avascular neurosis were equally pronounced among males and females, and on average were 50% higher in females than in males. The relative hazard of OA was also on average 20% higher in females than in males. There was a significant interaction between sex and the indication for THA (*p* < 0.001). The interaction between the relative hazards of post-operative complications and gender showed that females had greater risks of developing AVN, OA, and trauma than males, except for “the other” complications category. For the gender effect, females with AVN as an indication for THA had a 27% higher risk of complication (hazard ratio (HR) 1.27, 95% CI 1.16–1.38) as compared with the males. In addition, females with OA as an indication for THA had a 6% higher risk of complication (hazard ratio (HR) 1.06, 95% CI 1.03–1.09) as compared with males (Figure 5a). For the race effect, black patients with OA as an indication for THA had a 21% higher risk of developing complications (hazard ratio (HR) 1.21, 95% CI 1.11–1.30) as compared with Hispanic patients (Figure 5b). Moreover, black patients with OA as an indication for THA were more likely than white patients to have a complication (HR 1.07, 95% CI 1.02–1.13, *p* < 0.007), while Hispanic patients with OA as an indication for THA were less likely than white patients to have a complication (HR 0.89, 95% CI 0.83–0.95, *p* < 0.001). When all hip complications were combined, complications occurred with higher frequencies during the first two months of THA and the highest risks of THA complications within the first 6 months (Figure 6). There were also direct relationships between worse survival and increased overall complications (Figure 7).

## 4. Discussion

Understanding how risk factors relate to specific complications and the incidence of post-operative complications after primary THA will help explain not only the mechanism by which risk increases, but also how risk can be reduced via novel approaches, surgical techniques, and implants. Similar results were seen across studies in terms of survivorship and the reoperation rates of post-operative complications of primary THA across the life span [20,21,22,23]. Although hospital-based administrative data lacked a comparator group, the SID health data offered more detailed clinical data. In this study, the incidence of post-operative complications following primary THA was examined over 10 years within community hospitals using an SID database. This large study is unique in its ability to longitudinally investigate the patients undergoing primary THA with post-operative complications over years. Our findings suggested that patients with primary THA who were 55 years old, female, and black, with OA as an indication of THA, have exposed a higher risk profile in the setting of primary THA.

Previous investigations on post-operative complications in patients undergoing primary THA have reported the 35-year trend to incorporate THA in rheumatoid arthritis management, in addition to the classic indication for inflammatory OA and periprosthetic fracture [24]. Halvorsen and colleagues reported that the most common reasons for primary THA failures to require revisions were aseptic loosening (52%), dislocation (9.3%), deep infection (5.1%), and periprosthetic fracture (2.5%) in northern Europe [25]. The study with 10-year follow-up intervals showed a trend toward worse implant survival rates in patients with OA-related osteoporosis compared to those with other indications for THA [26]. Similarly, the Kaiser Permanente total joint replacement registry study demonstrated that the most common indication for primary THA was OA [27]. These findings are not surprising since arthroplasty is the surgical procedure of choice for OA in the knee and shoulder [28]. Our findings agree that the primary THA provide the reliable outcomes for patients suffering from end-stage degenerative hip OA [29]. Results from regional hospitals showed that the most common indications for primary THA are OA (65.5%), followed by AVN (22.1%) [30]. The length of hospital stays among patients in this study was slightly lower than those reported in previous studies by Wolford et al. (3.5 days) [31] and Bristol (4.23 days) [32]. This might be due to the fact that LOS following primary THA decreased over time, while the revision THA has a longer surgery time, more expensive prostheses, longer length of stay, and higher rates of complications and burden on the healthcare system [18]. The most common indications for THA were degenerative hip OA, followed by post-traumatic arthritis, and hip AVN, as reported in various geographical populations [33,34]. Interestingly, the incidence of AVN following primary THA varies significantly across different countries. Osteonecrosis, also known as AVN, results from the loss of blood supply to the bone, and its known risk factors include hip dislocations and femoral neck fractures following traumatic events or resulting from non-traumatic causes, which may eventually lead to the bone’s collapse and result in secondary OA [35]. In the United Kingdom, AVN is the third most common reason for primary THA in patients under 50 years old [36]. Consistent with our findings, the Defense Medical Epidemiology Database (DMED) showed that increased age and black race have the most significant influence on the development of AVN in the US military [37].

AVN commonly occurs in middle-aged males with recognized risk factors, such as alcohol use, steroid use, a history of smoking, and coagulation disorder, and may result in lower survival rates for a 10-year follow-up, consistent with a recent scoping review by Negm and colleagues [18]. Compared to those with OA, AVN patients have increased 30-day and long-term complication rates. In the short term, AVN patients have increased rates of readmission, surgical site infection, and medical complications [33]. In a 10-year follow-up, Ancelin and colleagues found that AVN was associated with increased revision and dislocation rates following THA [38]. The observed difference for increased readmission rates cannot be explained by traumatic or atraumatic AVN in the SID database, and future research is warranted in order to identify the modifiable factors of post-operative complications that account for the multifactorial risk factors of primary THA.

The incidence of post-operative complications after primary THA is particularly important for patients that are likely to have post-operative complications, especially females and those of black ethnicity. Our data show that these cohorts are sequelae of mechanical complications or thromboembolism. Population-based studies with early outcome data for THA were limited because most studies were completed at a single institution with a limited number of patients [39]. The current literature demonstrates trends to incorporate THA in fracture management, in addition to the classic indication for inflammatory OA. Studies with longer follow-up intervals have shown a trend towards worse implant survival rates in patients with OA compared to those with other indications for THA [40,41]. Prior database studies reported a 30-day readmission rate of 2.95% for outpatients and 3.21% for inpatients, and the 90-day complication rate for all adverse events was 3.8% [42,43]. Our periprosthetic joint infection rate of 1.02% was similar to that of the Medicare population, which was reported to range from 0.89 to 0.98% [7]. Thromboembolic complications were found to be 2.05% compared to 1.28% in an outpatient THA group [44,45]. Recent studies developed a predictive model of revision for dislocation within the first two years after primary THA using logistic regression and elastic net [45,46]. Our findings found a similar mechanical complication rate, which includes dislocation rates that were previously reported to be roughly 2–3% [46].

There are several limitations in this study. The time to the post-operative complication was provided in the linkage datasets; however, for patients who were administratively censored (e.g., the follow-up time ended due to a lack of additional data for that state), the time between the provided admission date and the end of the final available year for that state was used. Although the datasets were relatively complete, a few variables had missing values; these included ZIP-code-based income quartile (25.2%), race (7.6%), and sex (0.9%). Rather than performing a case-wise deletion of the records with missing values, we employed a statistical imputation technique to fill in missing datasets, which had previously been implemented in longitudinal clinical data [47]. Because of the state-specific nature of the SID, patients who moved between states within the study could not be linked to their initial THA. The potential for the absent or incorrect coding of relevant information and a lack of nuanced clinical details inherently limited the SID databases. Therefore, the events occurring outside the hospital could not be followed or analyzed. We acknowledged that such a methodology may underestimate the rate of adverse outcomes. Our study lacked a comparable non-THA cohort. However, the present study made comparisons with studies and registries published within the region to minimize the effect of regional variations on the conclusions.

## 5. Conclusions and Future Directions

The current study suggests that the most common indication is OA in elders with a primary THA. Patients with AVN have increased long-term risks of revision surgery compared to patients with other indications. Females and those of black ethnicity showed the greatest risks of THA complications compared to males and those of other ethnicities. The probability of post-operative complications increased rapidly during the first three months and steadily increased from 3 months to 10 years. Numerous pre-operative risk factors have been identified in this study, including advanced age, gender, race, and geographical locations. The occurrence of post-operative complications following primary THA were greatly decreased at the age of 55 and above and was associated with an increased LOS in the first week. Data from our large study can be used to understand risk of post-operative complications and readmissions after THA. Our study also provides data on risk factors associated with these complications.

## Figures and Tables

**Table 1:** Demographics of patients experiencing post-operative complications that required surgery.

	Mechanical Complication (*n* = 18,506)	Infection Due to Prosthetic Device (*n* = 5493)	Other (*n* = 3613)	Thromboembolism (*n* = 10,881)	Overall (*n*= 35,005)
**Age**					
Mean (SD)	65.4 (12.4)	64.0 (12.5)	62.5 (12.5)	69.5 (12.1)	66.3 (12.5)
Median [Q25, Q75]	66.0 [57.0, 75.0]	64.0 [56.0, 73.0]	63.0 [54.0, 72.0]	71.0 [62.0, 79.0]	67.0 [58.0, 76.0]
**Sex**					
Female	11,452 (61.9%)	2840 (51.7%)	2055 (56.9%)	6125 (56.3%)	20,488 (58.5%)
Male	6952 (37.6%)	2634 (48.0%)	1534 (42.5%)	4729 (43.5%)	14,361 (41.0%)
Other					
**Race**					
White	15,327 (82.8%)	4284 (78.0%)	2906 (80.4%)	8621 (79.2%)	28,325 (80.9%)
Hispanic	703 (3.8%)	254 (4.6%)	145 (4.0%)	532 (4.9%)	1501 (4.3%)
Black	1056 (5.7%)	436 (7.9%)	269 (7.4%)	948 (8.7%)	2410 (6.9%)
Other	482 (2.6%)	166 (3.0%)	91 (2.5%)	324 (4.2%)	970 (2.8%)
Large metropolitan area	10,554 (57.0%)	3058 (55.7%)	2037 (56.4%)	6667 (61.3%)	20,336 (58.1%)
Small metropolitan area	6175 (33.4%)	1852 (33.7%)	1256 (34.8%)	3296 (30.3%)	11,395 (32.6%)
Rural	1754 (9.5%)	575 (10.5%)	316 (8.7%)	908 (8.3%)	3230 (9.2%)
**LOS**					
Mean (SD)	3.87 (2.6)	4.22 (3.47)	3.8 (2.37)	4.48 (3.89)	4.06 (3.11)
Median [Q25, Q75]	3.00 [3.00, 4.00]	3.00 [3.00, 4.00]	3.00 [3.00, 4.00]	3.00 [3.00, 5.00]	3.00 [3.00, 4.00]
**Readmission Score**					
Mean (SD)	7.40 (9.83)	8.76 (11.1)	6.74 (9.38)	8.63 (10.7)	7.75 (10.2)
Median [Q25, Q75]	5.00 [0, 12.0]	6.00 [0, 14.0]	4.00 [0, 11.0]	7.00 [0, 14.0]	5.00 [0, 13.0]

**Table 2:** Patients’ demographics and indications for primary THA.

	Avascular Necrosis (*n* = 1686)	Osteoarthritis (*n* = 16,477)	Other (*n* = 956)	Trauma (*n* = 1492)	Overall (*n* = 20,611)
**Sex**					
Female	8668 (51.5%)	9664 (58.7%)	590 (61.7%)	955 (64.0%)	12,077 (58.6%)
Male	805 (47.7%)	6685 (40.6%)	364 (38.1%)	525 (35.2%)	8379 (40.7%)
Other					
**Race**					
White	1236 (73.3%)	13,791 (83.7%)	722 (75.5%)	1236 (82.8%)	16,985 (82.4%)
Hispanic	91 (5.4%)	545 (3.3%)	79 (8.3%)	61 (4.1%)	776 (3.8%)
Black	208 (12.3%)	881 (5.3%)	55 (5.8%)	62 (4.2%)	1206 (5.9%)
Other	49 (2.9%)	384 (2.3%)	41 (4.3%)	40 (2.7%)	514 (2.5%)
**Age**					
18–45	330 (19.6%)	653 (4.0%)	149 (15.6%)	84 (5.6%)	1216 (5.9%)
45–65	944 (56.0%)	7046 (42.8%)	399 (41.7%)	478 (32.0%)	8867 (43.0%)
65–80	315 (18.7%)	6954 (42.2%)	267 (27.9%)	549 (36.8%)	8085 (39.2%)
80+	92 (5.5%)	1786 (10.8%)	140 (14.6%)	373 (25.0%)	2391 (11.6%)

## Data Availability

The datasets generated and/or analyzed during the current study are not available due to data use agreement from the vendor but are available from the corresponding author (N.B.J., MD, MSPH) upon reasonable request.

## Appendix A

Mechanical complications were identified using diagnostic codes: unspecified mechanical complication of internal orthopedic device, implant and graft; mechanical loosening of prosthetic joint; dislocation of prosthetic joint; prosthetic joint implant failure; peri-prosthetic fracture around prosthetic joint; peri-prosthetic osteolysis; articular bearing surface wear of prosthetic joint; other mechanical complications of prosthetic joint implant; or other mechanical complication of other internal orthopedic device, implant, and graft. Thromboembolism codes were used to identify the following: iatrogenic pulmonary embolism; other pulmonary embolism (artery or vein); venous embolism and thrombosis of unspecified deep vessels of lower extremity; deep vessels of proximal lower extremity; deep vessels of distal lower extremity; other specified veins; or unspecified site.

**Table A1:** ICD 9 codes with corresponding complications following total hip arthroplasty.

Codes	Group		Description
41511	Thromboembolism		Pulmonary embolism iatrogenic
41519	Thromboembolism		Other pulmonary embolism (artery or vein)
45340	Thromboembolism		Venous embolism and thrombosis of unspecified deep vessels of lower extremity
45341	Thromboembolism		Venous embolism and thrombosis of deep vessels of proximal lower extremity
45342	Thromboembolism		Venous embolism and thrombosis of deep vessels of distal lower extremity
4538	Thromboembolism		Venous embolism and thrombosis of deep vessels of other specified veins
4539	Thromboembolism		Venous embolism and thrombosis of deep vessels of unspecified site
99640	Mechanical complications		Unspecified mechanical complication of internal orthopedic device, implant, and graft
99641	Mechanical complications	Aseptic loosening	Mechanical loosening of prosthetic joint
99642	Mechanical complications	Dislocation	Dislocation of prosthetic joint
99643	Mechanical complications		Prosthetic joint implant failure
99644	Mechanical complications	Fracture	Peri-prosthetic fracture around prosthetic joint
99645	Mechanical complications	Osteolysis	Peri-prosthetic osteolysis
99646	Mechanical complications	Wear	Articular bearing surface wear of prosthetic joint
99647	Mechanical complications		Other mechanical complications of prosthetic joint implant
99649	Mechanical complications		Other mechanical complication of other internal orthopedic device, implant, and graft
99666	Infection due to prosthetic device		Infection of inflammatory reaction due to internal joint prosthesis
99677	Other complications		Other complications of internal (biological) (synthetic) prsothetic device, implant, and graft due to internal joint prosthesis
2851	Bleeding		Acute posthemorrhagic anemia
99811	Bleeding		Hemorrhage complicating a procedure
99812	Bleeding		Hematoma complicating a procedure
99830	Wound problem		Disruption of wound, unspecified
99831	Wound problem		Disruption of internal operation (surgical) wound
99832	Wound problem		Disruption of external operation (surgical) wound
99851	Wound problem		Infected postoperative seroma
99859	Wound problem		Other postoperative infection
9986	Wound problem		Persistent postoperative fistula
99883	Wound problem		Non-healing surgical wound
9560	Nerve injury		Injury to sciatic nerve
90250	Vessel injury		Injury to iliac vessel, unspecified
90253	Vessel injury		Injury to iliac artery
90254	Vessel injury		Injury to iliac vein
90259	Vessel injury		Injury to iliac blood vessels, other
9040	Vessel injury		Injury to common femoral artery
9041	Vessel injury		Injury to superficial femoral artery
9042	Vessel injury		Injury to femoral veins
